# Development of Attention and Accuracy in Learning a Categorization Task

**DOI:** 10.3389/fpsyg.2021.544135

**Published:** 2021-02-02

**Authors:** Leonora C. Coppens, Christine E. S. Postema, Anne Schüler, Katharina Scheiter, Tamara van Gog

**Affiliations:** ^1^LEAD Graduate School & Research Network, University of Tübingen, Tübingen, Germany; ^2^Multiple Representations Lab, Leibniz-Institut für Wissensmedien, Tübingen, Germany; ^3^Department of Education, Utrecht University, Utrecht, Netherlands

**Keywords:** categorization, attention, learning, eye movements, eye-tracking

## Abstract

Being able to categorize objects as similar or different is an essential skill. An important aspect of learning to categorize is learning to attend to relevant features (i.e., features that determine category membership) and ignore irrelevant features of the to-be-categorized objects. Feature variability across objects of different categories is informative, because it allows inferring the rules underlying category membership. In this study, participants learned to categorize fictitious creatures (i.e., aliens). We measured attention to the aliens during learning using eye-tracking and calculated the attentional focus as the ratio of attention to relevant versus irrelevant features. As expected, participants’ categorization accuracy improved with practice; however, in contrast to our expectations, their attentional focus did not improve with practice. When computing the attentional focus, attention to the aliens’ eyes was disregarded, because while eyes attract a lot of attention, they did not vary across aliens (non-informative feature). Yet, an explorative analysis of attention to eyes suggested that participants’ attentional focus did become somewhat more efficient in that over time they learned to ignore the eyes. Results are discussed in the context of the need for instructional methods to improve attentional focus in learning to categorize.

## Introduction

The ability to categorize (i.e., to put people or things into groups with the same features) is an important skill for people. For example, it is crucial for people to learn, which types of food are safe and poisonous, and to classify food into groups such as edible and inedible. Categorization also plays an important role in many formal learning domains such as biology, psychology, physics, and chemistry. Not surprisingly, people’s ability to learn to categorize information has been well researched (e.g., Malt, 1995; Maddox et al., 2003; Birnbaum et al., 2013; Carvalho and Goldstone, 2017; for an overview, see Richler and Palmeri, 2014).

Different accounts of categorization exist in the literature (Erickson and Kruschke, 1998; Ashby and Maddox, 2005). One account is that categorization is based on rules and definitions. For example, if an animal lays eggs and has feathers and a beak, it is a bird. Another is that objects are categorized based on their similarity to other members of the category or to a prototype. For example, if an animal looks, swims, and quacks like a duck, it is a duck. The categorization task that we used in the present study was rule-based. Therefore, we focused on the first way of categorization, and we investigated the role of attention in learning rule-based categorization. Efficient rule-based categorization requires learners to attend to certain features and ignore others, as learners have to identify which features are associated with membership of a particular category (i.e., relevant for categorization; henceforth referred to as relevant features) and which features are irrelevant for categorization (henceforth referred to as irrelevant features). For example, geography students need to learn how to categorize rocks. When categorizing rocks, a student may look at the size of the rock to categorize it, even though rock size does not give much information about the type of rock (i.e., irrelevant feature), contrary to, for example, its color (i.e., relevant feature).

When learning to categorize objects into different categories, features that are the same for all objects should be discarded since they are not informative for category membership. On the other hand, features showing variability across objects may be informative in that they potentially indicate meaningful differences between categories and can hence be used to infer the rules underlying categorization (i.e., which features are either relevant or irrelevant). It is well-known that many people struggle with directing their attention to task-relevant features, especially in an unfamiliar task (Haider and Frensch, 1999; Hegarty et al., 2010; Jarodzka et al., 2010). A useful way to measure this process is eye-tracking. For instance, Rehder and Hoffman (2005) used an eye-tracking experiment to measure development of attention during category learning. They observed that participants indeed increasingly focused on relevant stimulus dimensions as they learned to categorize the stimuli.

Blair et al. (2009a) also conducted an eye-tracking study, but they focused on attention allocation immediately *after* learning a categorization task. In their study, participants had to learn to categorize fictitious microorganisms. These stimuli were designed specifically to study participants’ ability to allocate attention to relevant features over time while ignoring irrelevant features. Blair et al. (2009a) concluded that after learning to correctly categorize the microorganisms, participants’ attention distribution improved: over time, more time was spent looking at relevant features compared to the irrelevant feature. This finding was replicated in further studies with the same stimulus materials (Blair et al., 2009b; McColeman et al., 2011). More broadly, the idea that learners optimize their attention to look more at relevant information and less at irrelevant information with practice is known as the information reduction hypothesis (Haider and Frensch, 1999).

The aim of the present study was to investigate the development of accuracy and attention distribution during and after categorization learning. We used categorization rules similar to those of Blair et al. (2009a), but with different materials which included a systematically manipulated, irrelevant feature. This additional feature increased the complexity of the task and allowed us to investigate attention for irrelevant features, also in the learning phase, in more detail. The stimuli we used were developed based on an existing alien categorization task used by Carvalho and Goldstone (2017).

In sum, we aimed to investigate the development of categorization accuracy and attention distribution while participants were learning the categorization task, and in a test phase, after having learned the categorization rules. We expected that over the course of the learning phase, participants’ accuracy would increase and they would spend more time looking at relevant compared to irrelevant features of the aliens, and that participants’ attention distribution would continue to improve in the test phase.

## Materials and Methods

### Participants and Design

This study consisted of a learning phase (200 trials in eight blocks of 25 trials) and a test phase (72 trials) to which participants proceeded when they had completed 24 consecutive trials in the learning phase correctly. An *a priori* power analysis in G*Power using an expected effect size of *f* = 0.25 and a required power of *β* = 0.90 indicated a minimal sample size of *N* = 20 in the learning phase and *N* = 30 in the test phase. Because we expected that approximately 40% of the participants would not reach the test phase (cf. Blair et al., 2009a) and to compensate for any other exclusions, we recruited 51 university students who participated voluntarily and received a compensation of 10 euros. As only eight participants learned to categorize the aliens, it was not possible to conduct a meaningful analysis of the test phase data. Hence, we decided only to analyze the development of categorization accuracy and attention in the learning phase. The eight participants who learned to classify the aliens were excluded from further analysis, because they did not complete all 200 learning trials. Another 15 participants had to be excluded (one who did not complete the learning phase, two with an eye-tracking calibration deviation larger than 1 degree, 11 who had less than seven valid trials in at least one 25-trial block, and one participant whose eye-tracking data file was lost due to a technical failure). This resulted in a final sample of 28 participants, 21 female, seven male, age 19–66 years, *M*_age_ = 24.71, *SD* = 8.91.

### Ethics Statement

The study was reviewed and approved by the Ethics Committee of the Leibniz-Institut für Wissensmedien, Tübingen. Participants were informed about the nature and goals of the study, the fact they could stop their participation at any time, and their right to retract their data. All participants gave their informed consent before starting the experiment.

### Apparatus and Materials

#### Eye Tracker

We measured participants’ eye movements using an SMI REDm eye-tracker (SMI, Teltow, Germany) sampling at 250 Hz. Participants were seated with their eyes approximately 50–70 cm from the screen. Stimuli were presented in a learning environment programmed in JavaScript specifically for this experiment, and eye movement data were analyzed using SMI BeGaze software (version 3.7).

#### Categorization Task

**Figure 1:**
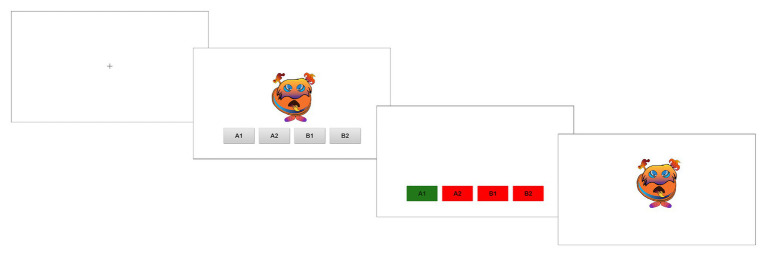
Order of screens in a trial in the learning phase.

The categorization task required classifying aliens into four categories. For an example of the stimuli, see Figure 1. All aliens had four body parts that varied in appearance: left antenna, right antenna, mouth, and feet. These features could each have one of two feature values (e.g., feet that are mainly blue or mainly orange, see Table 1), creating 2^4^ = 16 different aliens in total. The correct categorization depended on the feature values. The value of Feature 1 indicated whether the alien belonged to category A or B. The value of Feature 2 indicated within main category A whether the alien belonged to subcategory A1 or A2. The value of Feature 3 indicated within main category B whether the alien belonged to subcategory B1 or B2 (see Table 2). So, to classify a given alien, participants first had to look at Feature 1 to decide which main category the alien was and then depending on the main category look at Feature 2 or 3 to determine the subcategory of the alien. This means that to classify a given alien, two features were relevant: either Feature 1 and 2 or Feature 1 and 3. Feature 4 did not give any information for categorization, but did vary between aliens. The assignment of alien body parts (left antenna, right antenna, mouth, or feet) to features (Feature 1, 2, 3, or 4) was counterbalanced between participants.

**Table 1:** Features with feature values.

Feature name	Feature value 1	Feature value 2
Left antenna	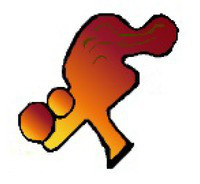	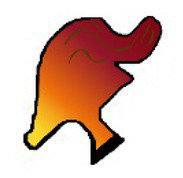
Right antenna	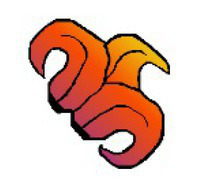	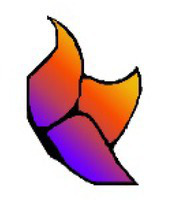
Mouth	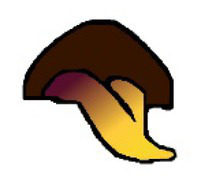	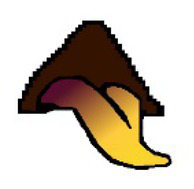
Feet	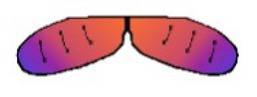	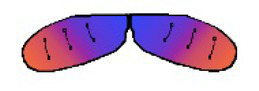

**Table 2:** Alien categories with corresponding feature values for categorization.

Category	Feature 1	Feature 2	Feature 3	Feature 4
A1	1	1	1 or 2	1 or 2
A2	1	2	1 or 2	1 or 2
B1	2	1 or 2	1	1 or 2
B2	2	1 or 2	2	1 or 2

*Features for which one feature value is given are relevant for categorizing a stimulus as a member of the category; features with two possible values are irrelevant for the category*.

The stimuli were presented in the middle of the screen on a monitor with a resolution of 1920 × 1,080 pixels. The on-screen dimensions of each alien were 7 × 8.5 cm (about 5.7 × 6.4° of visual angle).

### Procedure

The experiment took place in sessions with 1–5 participants in an eye-tracking lab and had a duration of approximately 50 min. First, participants filled out the informed consent form and a demographic questionnaire (gender, age, and main study subject) on paper. Then, after calibration of the eye-tracking equipment, participants read the following instructions:

“Recently a new planet with an alien population was discovered. The aliens belong to four different families (A1, A2, B1, B2), which differ in the visual appearance of the aliens. It is your task to find out which alien belongs to which family. The membership of an alien to a family is determined solely by the visual characteristics of an alien.

Next, you will first see a page with a cross. Look at the center of the cross. Please press the space bar to go to the next page. Then an alien appears on the screen with four answer boxes representing the different families. By clicking on the corresponding box, you decide to which family the alien belongs. Then click “Next” and you will get feedback on whether your decision was correct or incorrect. Then press the space bar to go to the next page. There you will get the opportunity to look at the alien again.

When you are ready to start the task, please press the space bar.”

Each trial started with a fixation cross in the middle of the screen, after which the first alien appeared. Four gray buttons with the four categories (A1, A2, B1, and B2) were shown below the alien. When the participant clicked one of the buttons, the alien disappeared and a feedback screen was shown in which the correct button was green and the incorrect buttons were red. When the participant pressed the space bar, the buttons disappeared and the same alien reappeared on the screen for restudy until the participant pressed the space bar again. The order of screens in each trial is shown in Figure 1. An alien of the same category (A1, A2, B1, and B2) never appeared twice consecutively.

The learning phase ended after 24 consecutive correct trials or after 200 trials in total, whichever came first. Participants who did not reach 24 consecutive correct trials were then debriefed and dismissed. The eight participants with 24 consecutive correct trials started the test phase. In the test phase, participants categorized 72 of the same aliens as in the learning phase without receiving feedback. After the test phase, they were debriefed and dismissed.

### Data Analysis

In SMI BeGaze, four areas of interest (AOIs) of equal size were created, one for each of the four features. We measured the total fixation duration on the AOIs on the choice screen per trial (fixation was defined as having a duration of at least 50 ms). Because the fixation cross was in the center of the screen, where the alien appeared, the first fixation in every trial was excluded. Valid trials were defined as trials with a duration of at least 200 ms (in order to exclude trials in which participants responded with only minimal processing of the image) and a maximum duration of the participant mean plus three standard deviations, and at least one fixation on any of the features, in which eye-tracking data was recorded during at least 80% of the trial duration. We excluded invalid trials from the eye-tracking analyses and computed averages over the remaining trials. Data from participants with less than seven valid trials in at least one 25-trial block were excluded entirely (11 participants, see section “Participants and Design”). Of the final sample of 28 participants, 11% of trials were excluded from the analyses.

#### Categorization Performance Measure

Categorization accuracy (correct or incorrect; 1 or 0 points) was summed per time block of 25 consecutive trials, which resulted in eight data (time) points per participant with a possible range of 0–25.

#### Attentional Focus Measure

The attentional focus measure was based on the attention optimization measure used by Blair et al. (2009a). This formula was adjusted to the current experiment because our stimuli had four features instead of three; we added the fourth feature to the formula. Therefore, for each trial we defined two features as relevant, and two features as irrelevant.

Attentional focus was measured by calculating the difference between the fixation duration on irrelevant features and the fixation duration on relevant features and dividing that difference by the sum of the fixation duration on irrelevant features and the fixation duration on relevant features:

$$\mathrm{Attentional focus}=\frac{\left(\mathrm{fix}.\mathrm{dur}.\mathrm{relevant}-\mathrm{fix}.\mathrm{dur}.\mathrm{irrelevant}\right)}{\left(\mathrm{fix}.\mathrm{dur}.\mathrm{relevant}+\mathrm{fix}.\mathrm{dur}.\mathrm{irrelevant}\right)}$$

The result ranges from −1 to 1. A score of −1 indicates the participant looked only at irrelevant features during a trial and a score of 1 indicates the participant looked only at relevant features. Attentional focus was averaged per participant per time block of 25 trials, which resulted in eight data (time) points per participant.

## Results

Descriptive statistics are shown in Table 3. Greenhouse-Geisser corrected degrees of freedom are reported for analyses in which the assumption of sphericity was violated. An alpha level of *α* = 0.05 was used for all analyses.

**Table 3:** Means (SD) of the dependent variables per time block in the learning phase.

Variable	Block 1	Block 2	Block 3	Block 4	Block 5	Block 6	Block 7	Block 8
Accuracy (0–25)	8.46 (3.45)	11.29 (4.32)	12.18 (4.18)	12.04 (4.36)	13.29 (4.63)	12.64 (4.89)	13.39 (4.96)	13.21 (4.98)
Attentional focus (−1–1)	0.245 (0.32)	0.237 (0.38)	0.219 (0.42)	0.263 (0.38)	0.288 (0.44)	0.322 (0.44)	0.273 (0.38)	0.298 (0.39)
Proportion attention to eyes (0–1)	0.236 (0.11)	0.177 (0.11)	0.170 (0.14)	0.181 (0.15)	0.164 (0.13)	0.178 (0.16)	0.151 (0.16)	0.148 (0.15)

### Categorization Accuracy

A repeated measures ANOVA with time block as a within-subjects factor showed a significant difference in categorization accuracy over time, *F*(3.88, 104.75) = 9.33, *p* < 0.001, $\eta_{p}^{2}$ = 0.257. There was a significant linear trend, *F*(1, 27) = 16.17, *p* < 0.001, $\eta_{p}^{2}$ = 0.375, indicating that, as expected, performance increased over time, and a significant quadratic trend, *F*(1, 27) = 13.48, *p* = 0.001, $\eta_{p}^{2}$ = 0.333, indicating that performance increased faster at the beginning of the learning phase than at the end.

An additional, explorative analysis of correctness of the main category (i.e., correctly categorizing an alien as A or B, regardless of the subcategory 1 or 2) yielded similar results: scores increased until the third block (a significant effect of Time on the main category correctness, *F*(4.00, 108.12) = 10.15, *p* < 0.001,$\;\eta_{p}^{2}$ = 0.237).^1^

### Attentional Focus

A repeated measures ANOVA with time block as a within-subjects factor showed no significant difference in attentional focus over time, *F*(3.53, 95.24) = 0.96, *p* = 0.424, $\eta_{p}^{2}$ = 0.034. A subsequent Bayesian analysis of the effect of time on attentional focus resulted in a Bayes factor of BF_10_ = 0.040, which indicates strong evidence for the null hypothesis. So in contrast to our expectation, attentional focus did not reliably increase over the course of the learning phase.

Additionally, we exploratively checked whether the proportion of fixations on Feature 1 (the feature that determines membership of the main category) increased in the course of the learning phase. If there was such an increase, this would suggest that although the attentional focus measure did not improve, participants did learn to focus more on the feature that was always relevant. However, there was no effect of time on proportion of time spent looking at the always relevant feature: *F*(3.42, 92.41) = 1.05, *p* = 0.382, $\eta_{p}^{2}$ = 0.037.

### Explorative Analysis

Next to the four changing features used in the attentional focus measure, the aliens all had one feature that did not change across aliens, but did draw a lot of attention: the eyes. Eyes are a highly salient feature for humans (e.g., Levy et al., 2013). In the initial analysis, we had not considered attention to the eyes, because they were not informative as they never changed for the aliens. However, given the fact that eyes tend to attract a lot of attention, one could argue that efficiency in learning the categorization should also be expressed in the degree to which our participants learned to ignore the eyes. Therefore, we also investigated the development of the proportion of time participants focused on the eyes in the course of the experiment (fixation duration on the eyes divided by fixation duration on all features; this measure ranges from 0 to 1, higher values indicating a higher fixation duration on the eyes compared to the other features). A repeated measures ANOVA on the proportion of time participants focused on the eyes with time block as a within-subjects factor showed a significant difference over time, *F*(3.40, 91.83) = 3.529, *p* = 0.014, $\eta_{p}^{2}$ = 0.116. There was a significant linear trend, *F*(1, 27) = 5.650, *p* = 0.025, $\eta_{p}^{2}$ = 0.173, indicating that time spent on looking at the eyes relative to the other features decreased over the course of the learning phase.

## Discussion

This study aimed to investigate the development of attentional focus during and after learning a categorization task. However, as most of our participants did not fully learn to categorize the aliens and we could not conduct the planned analyses over the test phase, we only analyzed the development of attention and accuracy during the learning phase. We investigated how task experience influenced attentional focus and categorization accuracy in learning the alien categorization task. Results show that participants were learning to categorize the aliens: categorization accuracy improved over time. However, there were no significant differences in attentional focus over the course of the learning phase. So while participants did become better at categorizing the aliens, this was not associated with increased focus on the relevant features while ignoring the irrelevant features.

To explain these findings, a closer look at other studies that investigated attention during categorization learning seems warranted. Rehder and Hoffman (2005) investigated the development of selective attention during categorization learning and found a gradual improvement. However, when they examined the individual participant data, it appeared that, for some participants, the attention shift to relevant features was in fact not gradual but abrupt. For those participants, the shift occurred in one or two trials, at a point soon after the participant had learned the categorization rule. After that point, no more categorization errors were made and the participant only focused on relevant information. Moreover, for all participants the attention shift, whether it was abrupt or more gradual, tended to occur only after a large reduction or even elimination of errors; hence, after learning the categorization rules. Blair et al. (2009a) did not find attention optimization before learning the categorization rules either. This could be an explanation for our results: none of the participants in our analysis learned the categorization rules. As none of the participants had made the attentional focus shift yet, we did not find a gradual attention shift.

In an additional exploratory analysis, we looked at attention to the eyes of the alien. Because the eyes were the same for every alien and hence not informative for the categorization, we did not include this area in our initial attentional focus measure. However, humans have a strong tendency to look at eyes, even in non-natural faces (Levy et al., 2013); a tendency that likely evolved because looking at the eyes and following the gaze of others can give us socially relevant information (Emery, 2000). Furthermore, the aliens’ gaze was directed straight at participants (i.e., direct gaze, rather than averted gaze), which facilitates eye contact; even virtual eye contact has been shown to influence attention and subsequent eye movements (Dalmaso et al., 2020). Moreover, at the start of the experiment participants did not know that the eyes were the same in all aliens. Therefore, one could argue that even though the eyes did not change, participants did have to learn to ignore the eyes in order to spend more time on the relevant features of the alien. Indeed, we found that in the course of the learning phase, time spent focusing on the eyes of the alien relative to the other features decreased. This suggests that participants did learn to ignore the eyes as they attempted to learn to categorize the aliens. These results add to recent findings on multimedia learning showing that with experience, learners can adjust their study behavior to ignore non-informative elements in a multimedia lesson (Rop et al., 2018).

That the majority of participants did not reach the test phase is an interesting result in its own. The learning phase of our experiment was similar to that of Blair et al. (2009a), in which 22 out of 38 participants reached the test phase. Our materials may have been more difficult for students to learn: whereas the underlying rule structure was similar, we used pictures of aliens instead of the bacteria used by Blair et al. (2009a). Our stimuli may have made learning more difficult because of pre-existing knowledge or assumptions about what a life form looks like. This finding underlines the value of conceptual replication attempts; when differences between tasks lead to different results, one should be careful not to generalize findings across different types of categorization tasks, without more research.

In all, this study has two important messages. First, it shows that it is important to replicate studies with different materials to gain understanding of the results and the implications. Second, categorization tasks can be complex, and it is difficult to ignore irrelevant features. Whereas our everyday experience tells us that we are effective in learning to distinguish between, for instance, apples and oranges, this does not necessarily mean that we are efficient in learning categorizations. Hence, an important question is how to speed up the process of learning. Therefore, future studies should focus on replications and on methods to improve people’s ability to ignore irrelevant features and look at relevant features.

## Data Availability Statement

The raw data supporting the conclusions of this article will be made available by the authors, without undue reservation.

## Ethics Statement

The studies involving human participants were reviewed and approved by the Ethics Committee of the Leibniz-Institut für Wissensmedien, Tübingen. The patients/participants provided their written informed consent to participate in this study.

## Author Contributions

CP, AS, KS, and TG contributed to the conception and design of the study. CP coordinated the data collection. LC performed the statistical analysis and wrote the first draft of the manuscript. All authors contributed to manuscript revision, read and approved the submitted version.

### Conflict of Interest

The authors declare that the research was conducted in the absence of any commercial or financial relationships that could be construed as a potential conflict of interest.

## Footnotes

^1^We would like to thank an anonymous reviewer for this suggestion.
